# Higher-Order Optimality Conditions in Set-Valued Optimization with Respect to General Preference Mappings

**DOI:** 10.1007/s11228-022-00627-2

**Published:** 2022-01-19

**Authors:** Anna Michalak, Marcin Studniarski

**Affiliations:** 1 Faculty of Economics and Sociology, University of Łódź, Rewolucji 1905 r. no. 41, 90-214 Łódź, Poland; 2 Faculty of Mathematics and Computer Science, University of Łódź, S. Banacha no. 22, 90-238 Łódź, Poland

**Keywords:** Set-valued optimization, Higher-order conditions, Preference mappings, Welfare economics, 49J52, 49J53, 91B15

## Abstract

We present higher order necessary conditions for a model of welfare economics, where the preference mapping has a star-shape property. We assume that the preferences can be satiable and can be described by an arbitrary preference set, without the use of utility functions. These conditions are formulated in terms of higher-order directional derivatives of multivalued mappings, and the variable domination structure is not given by cones.

**Mathematics Subject Classification (2010)** 49J52 · 49J53 · 91B15

## 1 Introduction

In economic equilibrium theory and in qualitative game theory, the behavior of economic agents or players is often determined by gereral preference mappings which do not necessarily lead to pre-order relations. As the authors of [8] state (p. 7), "All one needs to assume is that the deciding agent in state $x$ is able to specify those states $P(x)$ which he prefers

✉ Anna Michalak
anna.michalak@uni.lodz.pl

Marcin Studniarski
marcin.studniarski@wmii.uni.lodz.pl

1 Faculty of Economics and Sociology, University of Łódź, Rewolucji 1905 r. no. 41, 90-214 Łódź, Poland

2 Faculty of Mathematics and Computer Science, University of Łódź, S. Banacha no. 22, 90-238 Łódź, Poland to $x$. The only order conditions on preferences which is needed is *irreflexivity* (meaning $x \notin P(x)$)."

Microeconomic theory often assumes a property of nonsatiation, which means that consumers always choose to have more than less of goods. This leads to the conclusion that there is no point of satiation. The question is: is this assumption always fulfilled? We can imagine a situation that if we have two cars, we do not need a third one, or we do not need a second home if we already have one. Recently, more and more works have appeared in the literature which assume that preferences can be satiable. Mas-Collel in [17] was one of the first who considered the compact consumption sets and satiable preferences. Although he introduced a weaker than Walrasian equlibrium notion, his work started the discussion about the existence of an equilibrium if we do not assume nonsatiable preferences. Werner in [20] proved the existence of a competitive equilibrium without assuming either local or global nonsatiation. Afterwards Allouch and Le Van in [1] and [2] provided a weaker nonsatiation assumption to ensure the existence of competitive equilibria (see also [18]). Eventually Won and Yannelis in [21] generalized all classical equilibrium results to allow for possibly satiable preferences.

In this paper we present necessary optimality conditions for a model of welfare economics, where the preferences of individual customers (agents) are described by arbitrary preference sets, without the use of utility functions. We investigate minimization problems with variable domination structure. The optimality conditions are formulated in terms of some higher-order directional derivatives of multivalued mappings. In contrast to [5] and [6], the variable domination structure is not given by cones, therefore, it has more possible economic applications – see the examples in Section 6 where bounded preference sets are used. The only assumption that we make about the preference mapping is that it has a star-shape property, which is quite natural in the context of considered economic models.

Although also the authors of [4] consider the welfare economics model with an arbitrary preference mapping, their necessary conditions for localized minimizers in set-valued optimization are obtained under asymptotic closedness property of preference sets at local minimizers. The assumption of asymptotic clodedness is rather restrictive as it excludes bounded sets as possible preference sets. The aim of this paper is to prove the necessary conditions for strict local minimizers of order $m$ and to apply the obtained conditions to a model of welfare economics, avoiding the asymptotic closedness condition.

In the literature, there are many papers dealing with second order optimality conditions in set-valued optimization (among others, [9, 11–15]). However, these authors consider minimization problems with respect to closed convex cones. In our paper, minimization is understood with respect to some preference maping $P$, which is not necessarily cone-valued. Moreover, the optimality conditions obtained in the papers quoted above are stated in terms of some second-order derivatives (contingent, tangential or asymptotic), which are different from the derivatives used here.

We propose the following structure for this paper. Section 2 prepares mathematical preliminaries, Section 3 presents necessary optimality conditions of order $m$ for a general set-valued optimization problem, where we use the vector approach to define minimality. In Section 4, we briefly discuss some results for the set approach. In Section 5 we apply the results of Section 3 to welfare economics. Finally, Section 6 contains two concrete examples of optimization problems in welfare economics where bounded preference sets appear in a natural way.

## 2 Preliminaries

We start with some definitions from set-valued analysis. Let $X$ and $Y$ be normed spaces. We will use the notation $F : X \rightrightarrows Y$ for a set-valued mapping, i.e., $F : X \to 2^Y$.

For any two subsets $A$ and $B$ of $Y$, the symbol $A + B$ will detote the *algebraic sum* of $A$ and $B$:

$$A + B := \{a + b : a \in A, b \in B\}.$$

In a similar way, we define the *algebraic difference* $A - B$. For any $y \in Y$, we will simply write $y + A$ instead of $\{y\} + A$; the same for $y - A$.

**Definition 1** The *graph* of a set-valued mapping $F : X \rightrightarrows Y$ is defined by

$$\text{graph} F := \{(x, y) \in X \times Y : y \in F(x)\}.$$

**Definition 2** A set-valued mapping $P : Y \rightrightarrows Y$ is called a *preference mapping* if $y \notin P(y)$ for all $y \in Y$.

**Definition 3** A preference mapping $P : Y \rightrightarrows Y$ is called *star-shaped at* $y$ if $]y, z[ \subset P(y)$ for all $z \in P(y)$, where $]y, z[$ is the open line segment joining $y$ and $z$.

*Remark 1* The condition that $P$ is star-shaped at $y$ means that, for each $\lambda \in ]0, 1[$, we have

$$\lambda P(y) + (1 - \lambda)y \subset P(y),$$

which is equivalent to

$$\lambda(P(y) - y) \subset P(y) - y. \tag{1}$$

**Definition 4** (a) The *contingent cone* to a set $M \subseteq X$ at $\bar{x} \in \text{cl} S$ is defined as follows (see [15, p. 113]):

$$K(M, \bar{x}) := \{v \in X : \exists h_n \to 0^+, \exists v_n \to v \text{ s.t. } \bar{x} + h_n v_n \in M, \forall n\}. \tag{2}$$

(b) We also define the *radial tangent cone* (see [15, p. 110]):

$$\tilde{K}(M, \bar{x}) := \{v \in X : \exists h_n \to 0^+ \text{ s.t. } \bar{x} + h_n v \in M, \forall n\}. \tag{3}$$

We will denote by $\mathcal{N}(\bar{x})$ the set of all neighborhoods of a point $\bar{x} \in X$, and $B_Y(y, \delta)$ will be the open ball in $Y$ with center $y$ and radius $\delta > 0$.

Let $X$ and $Y$ be two normed spaces, and let $F : X \rightrightarrows Y$ be a set-valued mapping. We consider a nonempty set $S \subseteq X$ and the following optimization problem:

$$\text{Minimize } F(x) \text{ subject to } x \in S, \tag{4}$$

where the minimization is understood with respect to a given preference mapping $P$.

**Definition 5** Let $\bar{x} \in S$, and let $m$ be a positive integer. We say that a pair $(\bar{x}, \bar{y}) \in \text{graph} F$ is a *strict local minimizer of order m* for $F$ over $S$ with respect to preference mapping $P : Y \rightrightarrows Y$ if the following two conditions hold:

(i) $F(\bar{x}) \cap P(\bar{y}) = \emptyset$;

(ii) there exist $\alpha > 0$ and $U \in \mathcal{N}(\bar{x})$ such that, for each $x \in S \cap U \backslash \{\bar{x}\}$, we have

$$(F(x) + \bar{y} - P(\bar{y})) \cap B_Y(\bar{y}, \alpha \|x - \bar{x}\|^m) = \emptyset. \tag{5}$$

*Example 1* Let $X = Y = \mathbb{R}$,

$$F(x) := \begin{cases} \{1\} & \text{for } x = 0, \\ [3, 4] & \text{for } x > 0, \end{cases}$$

$S = [0, +\infty[$, $P(\bar{y}) = ]\bar{y}, \bar{y}+1]$. Then $(\bar{x}, \bar{y}) = (0, 1)$ is a strict local minimizer of arbitrary order $m \geq 1$ for $F$ over $S$ with respect to preference mapping $P$.

*Remark 2* Flores-Bazán and Jiménez in [7, Def. 3.2] have defined a *$\phi$-strict local minimizer for $F$ over $S$*. In their definition, the minimization is performed with respect to a proper convex cone $D$. Let us note that our condition (5) becomes condition (3.2) in [7] if we take $D := \bar{y} - P(\bar{y})$ (which is not necessarily a cone) and $\phi(t) := t^m$. Concerning condition (i), we have

$$\begin{aligned} F(\bar{x}) \cap P(\bar{y}) \ &= \ \emptyset \Leftrightarrow F(\bar{x}) \cap (\bar{y} - D) = \emptyset \Leftrightarrow (F(\bar{x}) - \bar{y}) \cap (-D) = \emptyset \\ &\Leftrightarrow \ (F(\bar{x}) - \bar{y}) \cap (-D \backslash \{0\}) = \emptyset, \end{aligned}$$

where the last equivalence follows from the condition $y \notin P(y)$. This means that (i) is equivalent to $\bar{y} \in \mathrm{Str}_D F(\bar{x})$ (see [7, Def. 2.1(b)]).

**Definition 6** (a) Let us introduce the following notation (see [16]) for the $m$-th order lower generalized directional derivative of $F$ at $(\bar{x}, \bar{y}) \in \mathrm{graph} F$:

$$\begin{aligned} \underline{d}^m F(\bar{x}, \bar{y})(u) \ := \ &\{v \in Y : \forall h_n \to 0^+, \forall u_n \to u, \exists v_n \to v \\ &\text{such that } \bar{y} + h_n^m v_n \in F(\bar{x} + h_n u_n) \text{ for all } n\}. \end{aligned} \tag{6}$$

(b) We also define

$$\begin{aligned} \underline{D}^m F(\bar{x}, \bar{y})(u) \ := \ &\{v \in Y : \forall h_n \to 0^+, \exists v_n \to v \\ &\text{such that } \bar{y} + h_n^m v_n \in F(\bar{x} + h_n u) \text{ for all } n\}. \end{aligned} \tag{7}$$

For $m = 1$, we will write $\underline{d} F$ and $\underline{D} F$ instead of $\underline{d}^1 F$ and $\underline{D}^1 F$, respectively.

The rest of this section contains a comparison of our Definition 5 with some notions introduced in [4].

**Definition 7** [4, Def. 3.1] Let $(\bar{x}, \bar{y}) \in \mathrm{graph} F$ with $\bar{x} \in S$ be given and let $P : Y \rightrightarrows Y$ be a given preference mapping. Then we say that:

(a) $(\bar{x}, \bar{y})$ is a *fully localized weak minimizer* for problem (4) if there exist $U \in \mathcal{N}(\bar{x})$ and $V \in \mathcal{N}(\bar{y})$ such that

$$F(S \cap U) \cap P(\bar{y}) \cap V = \emptyset \text{ with } F(S \cap U) := \bigcup \{F(x) : x \in S \cap U\}. \tag{8}$$

(b) $(\bar{x}, \bar{y})$ is a *fully localized strong minimizer* for problem (4) if there exist $U \in \mathcal{N}(\bar{x})$ and $V \in \mathcal{N}(\bar{y})$ such that

$$\mathrm{graph} F \cap (S \times \mathrm{cl} P(\bar{y})) \cap (U \times V) = \{(\bar{x}, \bar{y})\}. \tag{9}$$

*Example 2* Let $X = Y = \mathbb{R}$,

$$F(x) = \begin{cases} \{0\} & \text{for } x < 0, \\ \{0, 1\} & \text{for } x = 0, \\ \{x^2 + 1\} & \text{for } x > 0, \end{cases}$$

$S = [0, +\infty[$, $P(y) = ]-\infty, y[$, $U = ]-0.5, 0.5[$ and $V = ]0.5, 1.5[$. Then $(\bar{x}, \bar{y}) = (0, 1)$ is a fully localized strong minimizer for problem (4). Consequently, it is also a fully localized weak minimizer for this problem (see [15, Remark 2.6.50]).

*Example 3* (see [15, Example 2.6.53]) Let $X = \mathbb{R}$, $Y = \mathbb{R}^2$,

$$F(x) \equiv \mathbb{R}^2 \backslash \text{int}\mathbb{R}^2_- = \{(y_1, y_2) : (y_1 \geq 0) \vee (y_2 \geq 0)\},$$

$S = \mathbb{R}$, $P(y) = y - \text{int}\mathbb{R}^2_+$, $U = ]-\varepsilon, \varepsilon[$ and $V = ]-\varepsilon, \varepsilon[\times]-\varepsilon, \varepsilon[$, where $\varepsilon > 0$ is arbitrary. Then $(\bar{x}, \bar{y}) = (0, (0, 0))$ is a fully localized weak minimizer for problem (4) but it is not a fully localized strong minimizer for this problem.

**Proposition 1** *Let $(\bar{x}, \bar{y}) \in graph F$ with $\bar{x} \in S$ and a preference mapping $P$ all be given as well as $m \geq 1$ arbitrary but fixed.*

(a) *If $(\bar{x}, \bar{y})$ is a strict local minimizer of order $m$ for $F$ over $S$ with respect to $P$, then $(\bar{x}, \bar{y})$ is a fully localized weak minimizer for $F$ over $S$.*

(b) *If $(\bar{x}, \bar{y})$ is a strict local minimizer of order $m$ for $F$ over $S$ with respect to $P$, and the condition*

$$\text{cl}P(\bar{y}) = P(\bar{y}) \cup \{\bar{y}\} \tag{10}$$

*holds, then $(\bar{x}, \bar{y}) \in \text{graph}F$ is a fully localized strong minimizer for $F$ over $S$.*

*Proof* (a) Let $U$ and $\alpha$ be selected according to condition (ii) of Definition 5. Suppose that $(\bar{x}, \bar{y})$ is not a fully localized weak minimizer for $F$ over $S$. Then there exist sequences $\{x_n\} \subset S$ and $\{y_n\} \subset Y$ such that $x_n \to \bar{x}$, $y_n \to \bar{y}$ and

$$y_n \in F(x_n) \cap P(\bar{y}) \text{ for all } n. \tag{11}$$

We now consider the following two cases:

*Case 1*. There exists an infinite subsequence $\{x_{n_k}\}$ of $\{x_n\}$ such that $x_{n_k} \neq \bar{x}$ for all $k$.

*Case 2*. We have $x_n = \bar{x}$ for sufficiently large $n$.

In case 1, we have

$$\bar{y} = y_{n_k} + \bar{y} - y_{n_k} \in (F(x_{n_k}) + \bar{y} - P(\bar{y})) \cap B_Y(\bar{y}, \alpha\|x_{n_k} - \bar{x}\|^m) \text{ for all } k, \tag{12}$$

where we have used that $y_{n_k} \in F(x_{n_k})$, $-y_{n_k} \in -P(\bar{y})$ as a result of (11) and $\bar{y} \in B_Y(\bar{y}, \alpha\|x_{n_k} - \bar{x}\|^m)$. Condition (12) contradicts (5) because $x_{n_k} \in S \cap U \backslash \{\bar{x}\}$ for sufficiently large $k$. In case 2, condition (11) implies $y_n \in F(\bar{x}) \cap P(\bar{y})$ for sufficiently large $n$, which contradicts condition (i) of Definition 5. The contradiction reached in both cases completes the proof of part (a).

(b) Select $U$ and $\alpha$ as in part (a). Suppose that $(\bar{x}, \bar{y})$ is not a fully localized strong minimizer for $F$ over $S$. Then, for each $n \in \mathbb{N}$, there exist sequences $\{x_n\} \subset S$ and $\{y_n\} \subset Y$ such that $x_n \to \bar{x}$, $y_n \to \bar{y}$,

$$(x_n, y_n) \neq (\bar{x}, \bar{y}) \text{ for all } n, \tag{13}$$

and

$$y_n \in F(x_n) \cap \text{cl}P(\bar{y}) \text{ for all } n. \tag{14}$$

We now consider the cases 1 and 2 as in part (a). In case 1, for each $k \in \mathbb{N}$, since $y_{n_k} \in \text{cl}P(\bar{y})$ and $x_{n_k} \neq \bar{x}$, we can find $p_{n_k} \in P(\bar{y})$ such that

$$\|y_{n_k} - p_{n_k}\| < \alpha\|x_{n_k} - \bar{x}\|^m.$$

Therefore,

$$\bar{y} + y_{n_k} - p_{n_k} \in (F(x_{n_k}) + \bar{y} - P(\bar{y})) \cap B_Y(\bar{y}, \alpha\|x_{n_k} - \bar{x}\|^m),$$

which contradicts (5) because $x_{n_k} \in S \cap U \backslash \{\bar{x}\}$ for sufficiently large $k$. Suppose now that case 2 holds. Due to (13), we have $y_n \neq \bar{y}$ for sufficiently large $n$. Hence, using (10) and (14), we deduce $y_n \in \mathrm{cl} P(\bar{y}) \backslash \{\bar{y}\} = P(\bar{y})$ (note that $\bar{y} \notin P(\bar{y})$ since $P$ is a preference mapping). We have thus verified that $y_n \in F(\bar{x}) \cap P(\bar{y})$ for sufficiently large $n$, which contradicts condition (i) of Definition 5. This completes the proof of part (b). □

## 3 Necessary Optimality Conditions Based on the Vector Approach

Let $F : X \rightrightarrows Y$ and $G : X \rightrightarrows Z$ be two set-valued mappings. The following set-valued optimization problem is similar to problem (SOP) formulated in [16, p. 286]. In our formulation, the ordering cone $K \subset Y$ is replaced by a preference mapping $P : Y \rightrightarrows Y$:

$$\begin{cases} \text{Minimize } F(x) \text{ subject to} \\ x \in S := \{u \in X : u \in M, G(u) \cap (-Q) \neq \emptyset\}, \end{cases} \tag{15}$$

where $M$ is a nonempty subset of $X$, $Q$ is a closed convex cone in $Z$ with nonempty interior, and the minimization is understood with respect to $P$. The following theorem is an analogue of [16, Theorem 3.2] for this situation. A related result was obtained in [19] for a simple set-valued optimization problem without functional constraints.

**Theorem 1** *We consider problem* (15). *Let* $\bar{x} \in S$, $(\bar{x}, \bar{y}) \in \mathrm{graph} F$ *and* $\bar{z} \in G(\bar{x}) \cap (-Q)$ *be given. Suppose that the preference mapping* $P : Y \rightrightarrows Y$ *is star-shaped at* $\bar{y}$. *Let* $(\bar{x}, \bar{y})$ *be a strict local minimizer of order m for F over S with respect to P, and let* $\alpha > 0$ *and* $U \in \mathcal{N}(\bar{x})$ *be such that, for each* $x \in S \cap U \backslash \{\bar{x}\}$, *condition* (5) *holds. Then the following condition:*

$$\underline{d}^m F(\bar{x}, \bar{y})(u) \cap (B_Y(0, \beta\|u\|^m) + P(\bar{y}) - \bar{y}) = \emptyset \tag{16}$$

*holds for* $\beta := \alpha/2^m > 0$ *and for all*

$$u \in K(M, \bar{x}) \cap \{v : \underline{d}G(\bar{x}, \bar{z})(v) \subset -\mathrm{int}Q\} \backslash \{0\}, \tag{17}$$

*where* $K(M, \bar{x})$ *is defined by formula* (2).

*Proof* Condition (5) is equivalent to

$$w - y \notin B_Y(0, \alpha\|x - \bar{x}\|^m), \text{ for all } x \in S \cap U \backslash \{\bar{x}\}, w \in F(x) \text{ and } y \in P(\bar{y}). \tag{18}$$

Suppose that (16) does not hold, then there exists

$$\bar{u} \in K(M, \bar{x}) \cap \{v : \underline{d}G(\bar{x}, \bar{z})(v) \subset -\mathrm{int}Q\} \backslash \{0\} \tag{19}$$

such that

$$\underline{d}^m F(\bar{x}, \bar{y})(\bar{u}) \cap (B_Y(0, \beta\|\bar{u}\|^m) + P(\bar{y}) - \bar{y}) \neq \emptyset. \tag{20}$$

Since $\bar{u} \in K(M, \bar{x})$, there exist sequences $\{h_n\} \subset ]0, \infty[$ and $\{u_n\} \subset X$ with $h_n \to 0$ and $u_n \to \bar{u}$ such that

$$x_n := \bar{x} + h_n u_n \in M. \tag{21}$$

Moreover, take any $z \in \underline{d}G(\bar{x}, \bar{z})(\bar{u})$, then, for the preceding sequences $\{h_n\}$ and $\{u_n\}$, there exists a sequence $\{z_n\} \subset Z$ with $z_n \to z$ such that

$$\bar{z} + h_n z_n \in G(x_n). \tag{22}$$

By the conditions $\underline{d}G(\bar{x}, \bar{z})(\bar{u}) \subset -\mathrm{int}Q$ and $z \in \underline{d}G(\bar{x}, \bar{z})(\bar{u})$, we have $z \in -\mathrm{int}Q$. Hence, $z_n \in -Q$ for sufficiently large $n$. Then, by the assumption $\bar{z} \in -Q$ and the fact that $Q$ is a convex cone, we obtain

$$\bar{z} + h_n z_n \in -Q. \tag{23}$$

Conditions (22) and (23) imply

$$G(x_n) \cap (-Q) \neq \emptyset. \tag{24}$$

By (19), we have $\bar{u} \neq 0$, therefore, $x_n \neq \bar{x}$ for sufficiently large $n$. This condition, together with (21) and (24), gives for sufficiently large $n$,

$$x_n \in S \cap U \backslash \{\bar{x}\}. \tag{25}$$

On the other hand, it follows from (20) that there exists $\bar{v} \in \underline{d}^m F(\bar{x}, \bar{y})(\bar{u})$ such that

$$\bar{v} \in B_Y(0, \beta\|\bar{u}\|^m) + P(\bar{y}) - \bar{y}. \tag{26}$$

Since $\bar{v} \in \underline{d}^m F(\bar{x}, \bar{y})(\bar{u})$, for the preceding sequences $\{h_n\}$ and $\{u_n\}$, there exists a sequence $\{v_n\} \subset Y$ with $v_n \to \bar{v}$ such that

$$\bar{y} + h_n^m v_n \in F(x_n). \tag{27}$$

Since the set $B_Y(0, \beta\|\bar{u}\|^m) + P(\bar{y}) - \bar{y}$ is open, it follows from (26) that

$$v_n \in B_Y(0, \beta\|\bar{u}\|^m) + P(\bar{y}) - \bar{y}$$

for sufficiently large $n$. There exist $w_n \in B_Y(0, \beta\|\bar{u}\|^m)$ and $d_n \in P(\bar{y}) - \bar{y}$ such that $v_n = w_n + d_n$. We may assume that $h_n^m \in ]0, 1[$, which gives, in view of (1), recalling that $P$ is star-shaped, $h_n^m d_n \in P(\bar{y}) - \bar{y}$, or equivalently,

$$\bar{y} + h_n^m d_n \in P(\bar{y}). \tag{28}$$

We also have

$$h_n^m w_n \in h_n^m B_Y(0, \beta\|\bar{u}\|^m) = B_Y(0, \beta\|h_n\bar{u}\|^m). \tag{29}$$

From the convergence $u_n \to \bar{u} \neq 0$, we obtain that

$$\|\bar{u}\| - \|u_n\| \leq \|\bar{u} - u_n\| \leq \|u_n\|$$

for sufficiently large $n$. Hence, $\|\bar{u}\| \leq 2\|u_n\|$, which leads to

$$\beta\|\bar{u}\|^m = \frac{\alpha}{2^m}\|\bar{u}\|^m \leq \alpha\|u_n\|^m. \tag{30}$$

By (30), we have the following inclusion:

$$B_Y(0, \beta\|h_n\bar{u}\|^m) \subseteq B_Y(0, \alpha\|h_n u_n\|^m) = B_Y(0, \alpha\|x_n - \bar{x}\|^m). \tag{31}$$

Using (27), (28), (29) and (31), we obtain

$$h_n^m w_n = (\bar{y} + h_n^m v_n) - (\bar{y} + h_n^m d_n) \in (F(x_n) - P(\bar{y})) \cap B(0, \alpha\|x_n - \bar{x}\|^m), \tag{32}$$

which contradicts (18) in view of (25). The proof is complete. □

*Remark 3* The authors of [6] have obtained some necessary optimality conditions for set-valued optimization problems with variable ordering structure. They are first-order conditions only and are formulated mainly in terms of Bouligand derivatives of multifunctions Note that the Bouligand derivative $D_B F(\bar{x}, \bar{y})$ defined in [6, Definition 2.9] is the same as $\underline{d}F(\bar{x}, \bar{y})$ in our notation. However, the optimality conditions obtained there have a different structure than the ones in Theorem 1. For example, [6, Theorem. 3.3] involves both the derivative of $F$ (the multifunction being minimized) and the derivative of $K$ (the cone-valued multifunction defining the variable ordering).

*Remark 4* In [16, Theorem 3.2] it is assumed that $Q$ is a nontrivial pointed cone. However, the proof of Theorem 1 is valid without this assumption. In particular, we can take $Q = Z$ to obtain the following corollary.

**Corollary 1** *Suppose that the assumptions of Theorem 1 are satisfied where problem (15) is replaced by the following one:*

$$\text{Minimize } F(x) \text{ subject to } x \in M \tag{33}$$

*(i.e., the constraint $G(x) \cap (-Q) \neq \emptyset$ does not exist). If $(\bar{x}, \bar{y})$ is a strict local minimizer of order m for F over M with respect to P, then condition (16) holds for $\beta := \alpha/2^m > 0$ and for all $u \in K(M, \bar{x})\backslash\{0\}$.*

*Proof* This follows from Theorem 1 where $G$ is arbitrary and $Q = Z$. □

The following theorem is an analogue of [16, Thm. 3.4].

**Theorem 2** *We consider problem (15). Assume that $\bar{x} \in S$, $(\bar{x}, \bar{y}) \in \operatorname{graph} F$ and $\bar{z} \in G(\bar{x}) \cap (-Q)$. Suppose that the preference mapping $P : Y \rightrightarrows Y$ is star-shaped at $\bar{y}$. Let $(\bar{x}, \bar{y})$ be a strict local minimizer of order m for F over S with respect to P, and let $\alpha > 0$ and $U \in \mathcal{N}(\bar{x})$ be such that, for each $x \in S \cap U\backslash\{\bar{x}\}$, condition (5) holds. Then*

$$\underline{D}^m F(\bar{x}, \bar{y})(u) \cap (B_Y(0, \beta||u||^m) + P(\bar{y}) - \bar{y}) = \emptyset, \tag{34}$$

*for $\beta := \alpha > 0$ and for any*

$$u \in \tilde{K}(M, \bar{x}) \cap \{v : \underline{D}G(\bar{x}, \bar{z})(v) \subset -\operatorname{int} Q\}\backslash\{0\}.$$

*Proof* Arguing as in the proof of Theorem 1, we obtain (18). Assume that the conclusion of Theorem 2 is false, then there exists

$$\bar{u} \in \tilde{K}(M, \bar{x}) \cap \{v : \underline{D}G(\bar{x}, \bar{z})(v) \subset -\operatorname{int} Q\}\backslash\{0\} \tag{35}$$

such that

$$\underline{D}^m F(\bar{x}, \bar{y})(\bar{u}) \cap (B_Y(0, \beta||\bar{u}||^m) + P(\bar{y}) - \bar{y}) \neq \emptyset. \tag{36}$$

Since $\bar{u} \in \tilde{K}(M, \bar{x})$, there exists a sequence $\{h_n\} \subset ]0, \infty[$ with $h_n \to 0$ such that

$$\tilde{x}_n := \bar{x} + h_n\bar{u} \in M. \tag{37}$$

Moreover, taking any $z \in \underline{D}G(\bar{x}, \bar{z})(\bar{u})$, we obtain from the definition of $\underline{D}G(\bar{x}, \bar{z})(\bar{u})$ that for preceding sequence $\{h_n\}$, there exists a sequence $\{z_n\} \subset Z$ with $z_n \to z$ such that

$$\bar{z} + h_n z_n \in G(\tilde{x}_n). \tag{38}$$

By the conditions $\underline{D}G(\bar{x}, \bar{z})(\bar{u}) \subset -\operatorname{int} Q$ and $z \in \underline{D}G(\bar{x}, \bar{z})(\bar{u})$, we have $z \in -\operatorname{int} Q$. Then $z_n \in -Q$ for sufficiently large $n$. Similarly as in the proof of Theorem 1, we obtain

$$G(\tilde{x}_n) \cap (-Q) \neq \emptyset. \tag{39}$$

By (35), we have $\bar{u} \neq 0$, therefore, $\tilde{x}_n \neq \bar{x}$ for sufficiently large $n$. This condition, together with (37) and (39), gives

$$\tilde{x}_n \in S \cap U\backslash\{\bar{x}\} \tag{40}$$

for sufficiently large $n$.

On the other hand, it follows from (36) that there exists $\bar{v} \in \underline{D}^m F(\bar{x}, \bar{y})(\bar{u})$ such that (26) holds. Since $\bar{v} \in \underline{D}^m F(\bar{x}, \bar{y})(\bar{u})$, for the preceding sequence $\{h_n\}$, there exists a sequence $\{v_n\} \subset Y$ with $v_n \to \bar{v}$ such that

$$\bar{y} + h_n^m v_n \in F(\tilde{x}_n). \tag{41}$$

It follows from (26) by the same argument as in the proof of Theorem 1 that there exist $w_n \in B_Y(0, \beta\|\bar{u}\|^m)$ and $d_n \in P(\bar{y}) - \bar{y}$ such that $v_n = w_n + d_n$, condition (28) holds, and

$$h_n^m w_n \in B_Y(0, \beta\|h_n\bar{u}\|^m) = B_Y(0, \alpha\|\tilde{x}_n - \bar{x}\|^m). \tag{42}$$

Now, using (41), (28) and (42), we obtain

$$h_n^m w_n = (\bar{y} + h_n^m v_n) - (\bar{y} + h_n^m d_n) \in (F(\tilde{x}_n) - P(\bar{y})) \cap B(0, \alpha\|\tilde{x}_n - \bar{x}\|^m).$$

This contradicts (18) in view of (40). □

**Definition 8** Let $A$ be a convex subset of $Y$, and let $\bar{y} \in A$ be given. We define the *(convex) normal cone* to $A$ at $\bar{y}$ as follows:

$$N(A, \bar{y}) := \{y^* \in Y^* : \forall y \in A,\ \langle y^*, y - \bar{y}\rangle \leq 0\}. \tag{43}$$

**Theorem 3** *We consider problem* (15). *Let* $\bar{x} \in S$, $(\bar{x}, \bar{y}) \in \text{graph}F$ *and* $\bar{z} \in G(\bar{x}) \cap (-Q)$ *all be given. Suppose that the set* $P(\bar{y}) \cup \{\bar{y}\}$ *is convex and* $\text{int}P(\bar{y}) \neq \emptyset$. *Define*

$$D := K(M, \bar{x}) \cap \{v : \underline{d}G(\bar{x}, \bar{z})(v) \subset -\text{int}Q\} \backslash \{0\} \tag{44}$$

*and suppose that the set*

$$C := \bigcup_{u \in D} \underline{d}^m F(\bar{x}, \bar{y})(u) \tag{45}$$

*is convex and satisfies the condition*

$$\lambda C \subseteq C \textit{ for all } \lambda > 0. \tag{46}$$

*If* $(\bar{x}, \bar{y})$ *is a strict local minimizer of order m for* $F$ *over* $S$ *with respect to* $P$, *then there exists* $y^* \in Y^* \backslash \{0\}$ *such that*

$$\langle y^*, y\rangle \geq 0,\ \forall y \in \underline{d}^m F(\bar{x}, \bar{y})(u),\ u \in D, \tag{47}$$

$$\langle y^*, y\rangle \leq 0,\ \forall y \in (P(\bar{y}) \cup \{\bar{y}\}) - \bar{y}, \tag{48}$$

$$\langle y^*, y\rangle < 0,\ \forall y \in \text{int}(P(\bar{y}) - \bar{y}) = \text{int}(P(\bar{y}) \cup \{\bar{y}\}) - \bar{y}. \tag{49}$$

*Condition* (48) *implies, in particular, that*

$$y^* \in N((P(\bar{y}) \cup \{\bar{y}\}) - \bar{y}, 0) = N(P(\bar{y}) \cup \{\bar{y}\}, \bar{y}). \tag{50}$$

*Proof* The convexity of $P(\bar{y}) \cup \{\bar{y}\}$ implies that the mapping $P$ is star-shaped at $\bar{y}$. Hence, the assumptions of Theorem 1 are satisfied, and we get that condition (16) holds for all $u \in D$. Therefore

$$C \cap \text{int}(P(\bar{y}) - \bar{y}) = \emptyset. \tag{51}$$

It follows from (51) and the Eidelheit separation theorem (see [10, Thm. 3.16]) that there exist $y^* \in Y^* \backslash \{0\}$ and $\gamma \in \mathbb{R}$ such that

$$\langle y^*, y\rangle \leq \gamma \leq \langle y^*, c\rangle,\ \forall y \in (P(\bar{y}) \cup \{\bar{y}\}) - \bar{y},\ c \in C, \tag{52}$$

$$\langle y^*, y\rangle < \gamma,\ \forall y \in \text{int}((P(\bar{y}) \cup \{\bar{y}\}) - \bar{y}). \tag{53}$$

Conditions (46) and (52) imply that we can replace $\gamma$ by 0 in (52)–(53), which gives the desired conclusion. □

**Corollary 2** *We consider problem* (33). *Then Theorem 3 holds with* $D$ *replaced by* $K(M, \bar{x}) \backslash \{0\}$.

## 4 Necessary Optimality Conditions Based on the Set Approach

In this section, we present necessary optimality conditions for set-valued optimization problems, using the set approach as described in [15, Section 2.6.2].

**Definition 9** We say that a preference mapping $P : Y \rightrightarrows Y$ is *translation invariant* if there exists a subset $K$ of $Y$ such that

$$P(y) = y - K \text{ for all } y \in Y. \tag{54}$$

The following definition is a generalization of [15, Def. 2.6.13].

**Definition 10** Let a preference mapping $P$ be defined by (54). For arbitrary nonempty sets $A, B \in 2^Y$, the *possibly less domination relation* $\preceq_K^p$ is defined by

$$A \preceq_K^p B :\Leftrightarrow (\exists a \in A, \exists b \in B,\ b - a \in K) \Leftrightarrow (\exists a \in A, \exists b \in B,\ a \in P(b)). \tag{55}$$

**Definition 11** Let $X$ and $Y$ be two normed spaces, and let $F : X \rightrightarrows Y$ be a set-valued mapping. We consider a nonempty set $S \subset X$. We say that a point $\bar{x} \in S$ is a *set-based strict local minimizer of order* $m$ for $F$ over $S$ with respect to preference mapping $P : Y \rightrightarrows Y$ if there exist $\alpha > 0$ and $U \in \mathcal{N}(\bar{x})$ such that there is no $x \in S \cap U \backslash \{\bar{x}\}$ satisfying

$$F(x) \preceq_K^p B_Y(F(\bar{x}), \alpha \|x - \bar{x}\|^m), \tag{56}$$

where

$$B_Y(A, r) := \bigcup_{a \in A} B_Y(a, r). \tag{57}$$

**Theorem 4** *Let a preference mapping $P : Y \rightrightarrows Y$ be translation invariant. Suppose that there exists $\bar{y} \in F(\bar{x})$ such that $F(\bar{x}) \cap P(\bar{y}) = \emptyset$. Let $m$ be a positive integer. If $\bar{x} \in S$ is a set-based strict local minimizer of order $m$ for $F$ over $S$ with respect to $P$, then the pair $(\bar{x}, \bar{y})$ is a strict local minimizer of order $m$ for $F$ over $S$ with respect to $P$.*

*Proof* Since $P$ is translation invariant, there exists a set $K \subset Y$ such that (54) holds. By assumption, we have

$$\exists U \in \mathcal{N}(\bar{x}), \forall x \in S \cap U \backslash \{\bar{x}\},\ F(x) \not\preceq_K^p B_Y(F(\bar{x}), \alpha \|x - \bar{x}\|^m),$$

which is equivalent to

$$\exists U \in \mathcal{N}(\bar{x}), \forall x \in S \cap U \backslash \{\bar{x}\}, \forall y \in F(x), \forall \hat{y} \in F(\bar{x}),\ y \notin B_Y(\hat{y}, \alpha \|x - \bar{x}\|^m) - K. \tag{58}$$

Taking $\hat{y} := \bar{y}$ in (58), we get

$$\exists U \in \mathcal{N}(\bar{x}), \forall x \in S \cap U \backslash \{\bar{x}\}, \forall y \in F(x),\ y \notin B_Y(\bar{y}, \alpha \|x - \bar{x}\|^m) - K,$$

or equivalently,

$$\exists U \in \mathcal{N}(\bar{x}), \forall x \in S \cap U \backslash \{\bar{x}\},\ (F(x) + K) \cap B_Y(\bar{y}, \alpha \|x - \bar{x}\|^m) = \emptyset.$$

However, it follows form (54) that $K = \bar{y} - P(\bar{y})$. Therefore, condition (ii) of Definition 5 holds. By the choice of $\bar{y}$, condition (i) is also satisfied, which completes the proof. □

**Theorem 5** *We consider problem (15). Let $\bar{x} \in S$ and $\bar{z} \in G(\bar{x}) \cap (-Q)$. Suppose that there exists $\bar{y} \in F(\bar{x})$ such that $F(\bar{x}) \cap P(\bar{y}) = \emptyset$. Let the preference mapping $P : Y \rightrightarrows Y$ be translation invariant and star-shaped at $\bar{y}$. If $\bar{x}$ is a set-based strict local minimizer of order $m$ for $F$ over $S$ with respect to $P$, then there exists $\beta > 0$ such that*

$$\underline{d}^m F(\bar{x}, \bar{y})(u) \cap (B_Y(0, \beta \|u\|^m) + P(\bar{y}) - \bar{y}) = \emptyset,$$

*for all*

$$u \in K(M, \bar{x}) \cap \{v : \underline{d} G(\bar{x}, \bar{z})(v) \subset -\text{int} Q\} \backslash \{0\}.$$

*Proof* This theorem follows immediately from Theorems 1 and 4. □

*Remark 5* A similar result can be obtained by combining Theorems 2 and 4. We omit its formulation as it is rather obvious.

## 5 Applications to Welfare Economics

Consider a normed commodity space $E$ and preference mappings $P_i : Z \rightrightarrows E$ of $n$ customers ($i = 1, ..., n$), where $Z := E^n$ . Following [4, pp. 115–116], we define a set-valued mapping $F : E^{m+1} \rightrightarrows Z$ by

$$F(y, w) := \left\{ z \in Z : w = \sum_{i=1}^{n} z_i - \sum_{j=1}^{m} y_j \right\} \tag{59}$$

and a constraint set $\Omega \subset E^{m+1}$ by

$$\Omega := \left( \prod_{j=1}^{m} S_j \right) \times W. \tag{60}$$

We assume that the production sets $S_1, \ldots, S_m$ and the set $W$ are convex.

*Remark 6* In this section, the letter $m$ has a different meaning than in Sections 2–4. It is not related to the order of differentiation. Instead, it is used to denote the number of firms, as in [4, p. 114]. We hope this will not lead to a confusion as we use only derivatives of order one here.

We consider the following multiobjective optimization problem:

$$\text{Minimize} \;\; F(y, w) \;\; \text{subject to} \;\; (y, w) \in \Omega, \tag{61}$$

where minimization is understood with respect to a preference mapping $L : Z \rightrightarrows Z$ of the form

$$L(z) := \prod_{i=1}^{n} P_i(z). \tag{62}$$

Observe that problem (61) can be reduced to problem (15) in which $P = L$, $M = \Omega$ and $G$ does not exist. In order to apply the theory of Section 3 for order one, we will compute $\underline{d}F((\bar{y}, \bar{w}), \bar{z})(y, w)$.

**Proposition 2** *Let* $((\bar{y}, \bar{w}), \bar{z}) \in graph F$ *and* $(y, w) \in E^{n+1}$. *Then*

$$\underline{d}F((\bar{y}, \bar{w}), \bar{z})(y, w) = F(y, w). \tag{63}$$

*Proof* By the definition of $\underline{d}F$ (see formula (6) for $m = 1$), we have

$$\begin{aligned} \underline{d}F((\bar{y}, \bar{w}), \bar{z})(y, w) \; &= \{v \in E^n : \forall t_k \to 0^+, \forall (y_k, w_k) \to (y, w), \exists v_k \to v, \forall k, \\ \bar{z} + t_k v_k \; &\in \; F(\bar{y} + t_k y_k, \bar{w} + t_k w_k)\}. \end{aligned} \tag{64}$$

"$\subseteq$": Assume that $v \in \underline{d}F((\bar{y}, \bar{w}), \bar{z})(y, w)$. It follows from (59) and (64) that, for all $k$,

$$\bar{w} + t_k w_k = \sum_{i=1}^{n} (\bar{z} + t_k v_k)_i - \sum_{j=1}^{m} (\bar{y} + t_k y_k)_j. \tag{65}$$

Since $((\bar{y}, \bar{w}), \bar{z}) \in \mathrm{graph} F$, we have

$$\bar{w} = \sum_{i=1}^{n} \bar{z}_i - \sum_{j=1}^{m} \bar{y}_j, \tag{66}$$

hence (65) can be simplified to

$$w_k = \sum_{i=1}^{n} (v_k)_i - \sum_{j=1}^{m} (y_k)_j. \tag{67}$$

Passing to the limit as $k \to \infty$ in (67), we get

$$w = \sum_{i=1}^{n} v_i - \sum_{j=1}^{m} y_j, \tag{68}$$

which means that $v \in F(y, w)$.

"$\supseteq$": Suppose that $v \in F(y, w)$, then (68) holds. Take any sequences $t_k \to 0^+$ and $(y_k, w_k) \to (y, w)$. Define the sequence $\{v_k\}$ by

$$(v_k)_i := v_i \quad \text{for } i = 1, ..., n-1, \tag{69}$$

$$(v_k)_n := w_k - \sum_{i=1}^{n-1} v_i + \sum_{j=1}^{m} (y_k)_j. \tag{70}$$

We can now verify that $v_k \to v$. Indeed, this convergence is trivial for the first $n-1$ components, and for the last one we get

$$(v_k)_n \to w - \sum_{i=1}^{n-1} v_i + \sum_{j=1}^{m} y_j = v_n,$$

where the last equality follows from (68). Moreover, observe that conditions (69)–(70) imply (67). Combining (66) and (67), we get (65) (for all $k$), which gives that $v \in \underline{d}F((\bar{y}, \bar{w}), \bar{z})(y, w)$. $\square$

We want to apply Corollary 2 to problem (61). Observe that, by (60) and the convexity of the sets $S_1, \ldots, S_m, W$, we have that

$$K(\Omega, (\bar{y}, \bar{w})) = \left( \prod_{j=1}^{m} K(S_j, \bar{y}_j) \right) \times K(W, \bar{w}) \tag{71}$$

(see [3], Chapter 4, formula (46)).

Further, we need to ensure that the set $C$ defined by (45) (with $D$ replaced by $K(\Omega, (\bar{y}, \bar{w})) \backslash \{(0, 0)\}$) is convex and satisfies (46). This is verified in the following proposition using the notations of the present problem (61).

**Proposition 3** *Suppose that the cones $K(S_1, \bar{y}_1), ..., K(S_m, \bar{y}_m), K(W, \bar{w})$ are nontrivial and pointed. Then the set*

$$\tilde{C} := \bigcup_{(y,w) \in K(\Omega,(\bar{y},\bar{w})) \backslash \{(0,0)\}} \underline{d}F((\bar{y}, \bar{w}), \bar{z})(y, w) = \bigcup_{(y,w) \in K(\Omega,(\bar{y},\bar{w})) \backslash \{(0,0)\}} F(y, w) \tag{72}$$

*is convex and satisfies the condition*

$$\lambda \tilde{C} \subseteq \tilde{C} \ \text{ for all } \ \lambda > 0. \tag{73}$$

*Proof* By an argument similar to [10, Lemma 1.11], it is sufficient to prove the following two conditions: (73) and

$$\tilde{C} + \tilde{C} \subseteq \tilde{C}. \tag{74}$$

To prove (74), let us take any $z^1, z^2 \in \tilde{C}$. Then, for $l = 1, 2$, there exist

$$(y^l, w^l) \in K(\Omega, (\bar{y}, \bar{w}))\backslash\{(0, 0)\} \tag{75}$$

satisfying

$$w^l = \sum_{i=1}^{n} z_i^l - \sum_{j=1}^{m} y_j^l. \tag{76}$$

Defining $\tilde{w} := w^1 + w^2$, $\tilde{z} := z^1 + z^2$, $\tilde{y} := y^1 + y^2$ and adding together the two equalities (76), we get

$$\tilde{w} = \sum_{i=1}^{n} \tilde{z}_i - \sum_{j=1}^{m} \tilde{y}_j. \tag{77}$$

Conditions (71) and (75) imply that

$$y_j^l \in K(S_j, \bar{y}_j),\ \ w^l \in K(W, \bar{w}),\ \ j = 1, ..., m,\ \ l = 1, 2, \tag{78}$$

and,

$$\text{for each } l \in \{1, 2\}, \text{ at least one of the elements } y_1^l, ..., y_m^l, w^l \text{ is nonzero.} \tag{79}$$

Since the cones in (78) are convex, we deduce that

$$\tilde{y}_j \in K(S_j, \bar{y}_j),\ \ \tilde{w} \in K(W, \bar{w}),\ \ j = 1, ..., m. \tag{80}$$

This, in view of (71), means that $(\tilde{y}, \tilde{w}) \in K(\Omega, (\bar{y}, \bar{w}))$. Moreover, $\tilde{z} \in F(\tilde{y}, \tilde{w})$ by (77). To complete the proof of (74), we need to verify that $(\tilde{y}, \tilde{w}) \neq (0, 0)$, that is,

$$\text{at least one of the elements } \tilde{y}_1, ..., \tilde{y}_m, \tilde{w} \text{ is nonzero.} \tag{81}$$

Taking into account (79), we consider two cases:

(a) There exist nonzero elements in the sequences

$$\{y_1^1, ..., y_m^1, w^1\} \text{ and } \{y_1^2, ..., y_m^2, w^2\} \tag{82}$$

appearing on the same positions.

(b) Condition (a) does not hold.

*Case* (a). We have, for example, $y_k^1 \neq 0 \neq y_k^2$ for some $k \in \{1, ..., m\}$. Then $\tilde{y}_k = y_k^1 + y_k^2 \neq 0$ because the cone $K(S_k, \bar{y}_k)$ is pointed (otherwise, we would have $y_k^1 = -y_k^2 \in K(S_k, \bar{y}_k) \cap (-K(S_k, \bar{y}_k)) = \{0\}$, a contradiction). Therefore, condition (81) is satisfied.

*Case* (b). There exists a nonzero element in one of the sequences (82) with zero on the same position in the other sequence. For example, let $y_k^1 \neq 0 = y_k^2$. Then $\tilde{y}_k = y_k^1 + y_k^2 = y_k^1 \neq 0$ and condition (81) is also true.

The proof of (73) follows easily from (72) and the fact that $K(S_j, \bar{y}_j)$ $(j = 1, \ldots, m)$ and $K(W, \bar{w})$ are cones. □

We shall now prove necessary optimality conditions for problem (61).

**Theorem 6** *Let* $(\bar{y}, \bar{w}) \in \Omega$ *and* $((\bar{y}, \bar{w}), \bar{z}) \in \text{graph}F$. *Suppose that the sets* $P_i(\bar{z}) \cup \{\bar{z}_i\}$ *are convex and* $\text{int}P_i(\bar{z}) \neq \emptyset$, $i = 1, ..., n$, *the sets* $S_1, \ldots, S_m, W$ *are convex and the cones* $K(S_1, \bar{y}_1), ..., K(S_m, \bar{y}_m), K(W, \bar{w})$ *are nontrivial and pointed. We consider problem* (61). *If* $((\bar{y}, \bar{w}), \bar{z})$ *is a strict local minimizer of order 1 for* $F$ *over* $\Omega$ *with respect to L, then there*

*exists* $z^* \in Z^* \backslash \{0\}$, $z^* = (z_1^*, ..., z_n^*)$, *where* $z_i^* \in E^*$, $i = 1, ..., n$, *such that the following three conditions are satisfied:*

(i) *The inequality*

$$\langle z^*, z \rangle = \sum_{i=1}^{n} \langle z_i^*, z_i \rangle \geq 0 \tag{83}$$

*holds for all* $z = (z_1, ..., z_n) \in F(y, w)$ *where*

$$(y, w) = ((y_1, ..., y_m), w) \in \left( \prod_{j=1}^{m} K(S_j, \bar{y}_j) \right) \times K(W, \bar{w}) \text{ and } (y, w) \neq (0, 0). \tag{84}$$

(ii) $z_i^* \in N(P_i(\bar{z}) \cup \{\bar{z}_i\}, \bar{z}_i)$, $i = 1, ..., n$.

(iii) *There exists* $i_0 \in \{1, ..., n\}$ *such that*

$$\langle z_{i_0}^*, z_{i_0} \rangle < 0 \text{ for all } z_{i_0} \in \text{int}(P_{i_0}(\bar{z}) - \bar{z}_{i_0}). \tag{85}$$

*Proof* First, we will show that the assumptions of Theorem 3 are satisfied for the particular case described in Corollary 2. We consider problem (33) where $F : E^{m+1} \rightrightarrows Z$ is defined by (59) and $M = \Omega$ is defined by (60). Let $(\bar{y}, \bar{w}) \in \Omega$ and $((\bar{y}, \bar{w}), \bar{z}) \in \text{graph}F$. We have

$$L(\bar{z}) \cup \{\bar{z}\} = \prod_{i=1}^{n} (P_i(\bar{z}) \cup \{\bar{z}_i\}). \tag{86}$$

Since the sets $P_i(\bar{z}) \cup \{\bar{z}_i\}$ are convex, the set $L(\bar{z}) \cup \{\bar{z}\}$ is also convex. Moreover, we have by (62) and the well-known property of topological interior,

$$\text{int}L(\bar{z}) = \text{int} \left( \prod_{i=1}^{n} P_i(z) \right) = \prod_{i=1}^{n} \text{int}P_i(z).$$

Since the sets $\text{int}P_i(z)$ are nonempty, the set $\text{int}L(\bar{z})$ is also nonempty. The set (45) has now the form (72), so it is convex by Proposition 3. Therefore, the conclusion of Theorem 3 holds where $D$ should be replaced by $K(\Omega, (\bar{y}, \bar{w})) \backslash \{(0, 0)\}$. This gives the following condition: there exists $z^* \in Z^* \backslash \{0\}$, $z^* = (z_1^*, ..., z_n^*)$, $z_i^* \in E^*$, such that

$$\langle z^*, z \rangle \geq 0, \ \forall z \in F(y, w), \ (y, w) \in K(\Omega, (\bar{y}, \bar{w})) \backslash \{(0, 0)\}, \tag{87}$$

$$\langle z^*, z \rangle \leq 0, \ \forall z \in (L(\bar{z}) \cup \{\bar{z}\}) - \bar{z}, \tag{88}$$

$$\langle z^*, z \rangle < 0, \ \forall z \in \text{int}(L(\bar{z}) - \bar{z}). \tag{89}$$

The implication (87) $\Rightarrow$ (i) holds by (71). From (86) and (88), we obtain

$$z^* \in N(L(\bar{z}) \cup \{\bar{z}\}, \bar{z}) = \prod_{i=1}^{n} N(P_i(\bar{z}) \cup \{\bar{z}_i\}, \bar{z}_i),$$

where the equality follows from the well-known product formula for normal cones (see e.g. [4, eq. (2.6)]). Hence, $z_i^* \in N(P_i(\bar{z}) \cup \bar{z}_i, \bar{z}_i)$ for $i = 1, ..., n$. Finally, we will verify (iii). Suppose there is no $i_0 \in \{1, ..., n\}$ satisfying (85). Then, for all $i \in \{1, ..., n\}$, there exists $z_i \in \text{int}(P_i(\bar{z}) - \bar{z}_i)$ such that $\langle z_i^*, z_i \rangle \geq 0$. Consequently, we have

$$\langle z^*, z \rangle = \sum_{i=1}^{n} \langle z_i^*, z_i \rangle \geq 0 \text{ and } z \in \text{int}(L(\bar{z}) - \bar{z}),$$

which contradicts (89). □

## 6 Examples

In this section, we present two concrete examples of problem (61). We show that Theorem 6 can be applied to both examples but not at all points $(\bar{y}, \bar{z}) \in \operatorname{graph} F$.

*Example 4* There are $m$ companies on the global pharmaceutical market which produce COVID -19 vaccines. Every company has limited production capabilities: the production volume of company $j$ for a given year belongs to some bounded set $S_j$ $(j = 1, ..., m)$. The governments of $n$ countries are applying for the purchase of these vaccines. Each country $i$ would like to vaccinate as many people as possible but not more than the size $d_i$ of adult population of this country. We assume that various vaccines can be used interchangeably in every age group. It can be assumed that every set $S_j$ is a closed interval of the form $S_j = [0, p_j]$, where $p_j$ is the maximum volume of production of company $j$ in a considered period of time. Suppose also that there is no initial supply of vaccines: $W = \{0\}$. Then formula (59) can be simplified to the form:

$$F(y) := \left\{ z \in Z : \sum_{i=1}^{n} z_i - \sum_{j=1}^{m} y_j = 0 \right\}, \tag{90}$$

where $F : \mathbb{R}^m \rightrightarrows Z$. The set $Z = \mathbb{R}^n$ is the set of consumption plans of the form $z = (z_1, ..., z_n)$, while minimization $F$ is considered on the set $S_1 \times ... \times S_m \subset \mathbb{R}^m$. The set of preferences of the $i$-th customer (country) has the form

$$P_i(z) := \{u_i \in \mathbb{R} : z_i < u_i \leq d_i\},$$

which means that every country wants to have as many vaccines as possible for itself, and it is not interested in the situation in other countries.

Let $(\bar{y}, \bar{z}) \in \operatorname{graph} F$. We consider the following two cases, which are interesting from the practical viewpoint:

(a) There exists $l \in \{1, ..., m\}$ such that $\bar{y}_l \in ]0, p_l[$ (that is, at least one company does not sell its whole possible production). Then $K(S_l, \bar{y}_l) = \mathbb{R}$, and we cannot apply Theorem 5 because this cone is not pointed.

(b) We have $\bar{y}_j = p_j$ for all $j \in \{1, ..., m\}$ (that is, the production level of each company is set to maximum, and all the vaccines are sold). The following sub-cases are of practical interest (we omit the discussion of the case where $\bar{z}_i = 0$ for some $i$):

($b_1$) There exists $r \in \{1, ..., n\}$ such that $\bar{z}_r = d_r$ (the demand of at least one country is fully satisfied). Then $P_r(\bar{z}) = \emptyset$, which violates the assumptions of Theorem 6.

($b_2$) We have $0 < \bar{z}_i < d_i$ for all $i \in \{1, ..., n\}$. Then $N(P_i(\bar{z}) \cup \{\bar{z}_i\}, \bar{z}_i) = ]-\infty, 0]$ for all $i$, and it is easy to see that conditions (ii) and (iii) of Theorem 6 are satisfied if and only if $z^* \neq 0$ and $z_i^* \leq 0$ for all $i$. Concerning condition (i), since $K(S_j, \bar{y}_j) = ]-\infty, 0[$ for all $j \in \{1, ..., m\}$, then by (90), conditions

$$z \in F(y), \;\; y \in \prod_{j=1}^{m} K(S_j, \bar{y}_j)$$

imply that

$$\sum_{i=1}^{n} z_i = \sum_{j=1}^{m} y_j \leq 0.$$

By taking $z_i^* = -1$ for all $i$, we get that

$$\langle z^*, z\rangle = \sum_{i=1}^{n} \langle z_i^*, z_i\rangle = -\sum_{i=1}^{n} z_i = -\sum_{j=1}^{m} y_j \geq 0,$$

and therefore condition (i) of the theorem holds in this case.

**Discussion** We now discuss the results of this example from the point of view of possible application. First, in case (a) our theorem cannot be used but the answer can easily be deduced from the conditions of the problem. Indeed, if the demand of each country is fully satisfied, then the corresponding production-consumption plan is optimal for customers (but not necessarily for producers). If this is not the case, then the plan is obviously not optimal and can be improved by buying more vaccines by some customers. For sub-case ($b_1$), Theorem 6 also gives no answer but generally such plans should be avoided. It is better to have some shortage of vaccines in each country as it is known that some people don't want to be vaccinated. Finally, in sub-case ($b_2$), our theorem gives a positive answer which means that the corresponding plan is a candidate for optimal solution.

*Example 5* In some country, there are two factories producing electric cars. These cars can be either class A or class B. Factory 1 has one production line which can be used to assemble cars of both classes, depending on customers' orders. The maximum production capacity of this line for a given year is 2000 cars. Factory 2 has two independent production lines: the first one is designed for class A cars, and the second one – for class B cars. The maximum production capacity of each line is 1000 cars. There is only one company (a car dealer) which can buy cars form the two factories and sell them to individual customers, at the same time providing service to them. However, due to restricted infrastructure (the number of existing workshops and charging stations), this company cannot distribute more than $d$ cars for a year. For simplicity, we assume that one unit means 1000 cars.

In this example, we have $Z = \mathbb{R}^2$. A feasible consumption plan $z \in Z$ for the dealer has the form $z = (z^1, z^2)$, where $z^1$ is the number of distrubuted cars of class A, and $z^2$ – of class B; every such plan must satisfy the restriction $z^1 + z^2 \leq d$. Hence, the preference mapping of the dealer is defined by

$$P(z) := \{u = (u^1, u^2) : u^i \geq z^i,\ i = 1, 2,\ u \neq z,\ u^1 + u^2 \leq d\}. \tag{91}$$

The production sets for factories 1 and 2 are defined, respectively, by

$$S_1 := \{y_1 = (y_1^1, y_1^2) : y_1^i \geq 0,\ i = 1, 2,\ y_1^1 + y_1^2 \leq 2\},$$
$$S_2 := \{y_2 = (y_2^1, y_2^2) : 0 \leq y_2^i \leq 1,\ i = 1, 2\}.$$

The mapping $F : \mathbb{R}^2 \times \mathbb{R}^2 \to \mathbb{R}^2$ (here it is single-valued) has now the form

$$F(y) := \{z \in \mathbb{R}^2 : z = y_1 + y_2\},\ \text{where } y = (y_1, y_2).$$

Because this example is illustrative, we we will not consider all possible cases but only several selected ones. Below we consider three different values of $d$.

(a) Let $d = 5$. We consider the following two sub-cases.

($a_1$) Suppose that $\bar{y} = (\bar{y}_1, \bar{y}_2)$, where $\bar{y}_1^1 \in ]0, 2[$, $\bar{y}_1^2 = 2 - \bar{y}_1^1$, and $\bar{y}_2 = (1, 1)$. We compute the contingent cones to the production sets:

$$K(S_1, \bar{y}_1) = \{(y_1^1, y_1^2) : y_1^1 + y_1^2 \leq 0\}, \tag{92}$$
$$K(S_2, \bar{y}_2) = \{(y_2^1, y_2^2) : y_2^i \leq 0,\ i = 1, 2\}. \tag{93}$$

It is easy to see that, for any $\bar{z} = \bar{y}_1 + \bar{y}_2$, $\bar{y}_j \in S_j$, $j = 1, 2$, we have

$$z^* = (z^{*1}, z^{*2}) \in N(P(\bar{z}) \cup \{\bar{z}\}, \bar{z}) \Leftrightarrow (z^{*i} \leq 0,\ i = 1, 2).$$

Let us take $z^* = (-1, -1)$. We now check the fulfillment of conditions conditions (i)–(iii) of Theorem 6.

Let $z = F(y) = y_1 + y_2$, where $y \neq 0$ and $y_j \in K(S_j, \bar{y}_j)$, $j = 1, 2$. In particular, we have by (92)–(93),

$$y_1^1 + y_1^2 \leq 0 \text{ and } z^i = y_1^i + y_2^i \leq y_1^i,\ i = 1, 2.$$

Consequently,

$$\langle z^*, z\rangle = -z^1 - z^2 \geq -y_1^1 - y_1^2 \geq 0,$$

so that conditions (i) and (ii) hold. Further, assuming that

$$z \in \operatorname{int}(P(\bar{z}) - \bar{z}) \subset \{(z^1, z^2) : z^i > 0,\ i = 1, 2\},$$

we have

$$\langle z^*, z\rangle = -z^1 - z^2 < 0,$$

and condition (iii) is also fulfilled.

($a_2$) Suppose now that $\bar{y} = (\bar{y}_1, \bar{y}_2)$, where $\bar{y}_1^1 \in ]0, 2[$, $\bar{y}_1^2 = 2 - \bar{y}_1^1$, and $\bar{y}_2 = (1, \bar{y}_2^2)$, $\bar{y}_2^2 \in ]0, 1[$. Then $K(S_1, \bar{y}_1)$ is given by (92) and

$$K(S_2, \bar{y}_2) = \{(y_2^1, y_2^2) : y_2^1 \leq 0\}. \tag{94}$$

We can observe that the algebraic sum $K(S_1, \bar{y}_1)+ K(S_2, \bar{y}_2)$ is equal to the whole space $\mathbb{R}^2$. Therefore, it is impossible to find a nonzero vector $z^* = (z^{*1}, z^{*2})$ with $z^{*i} \leq 0$, $i = 1, 2$, which would satisfy $\langle z^*, z\rangle \geq 0$ for all $z \in K(S_1, \bar{y}_1) + K(S_2, \bar{y}_2)$. This means that Theorem 6 excludes such points $\bar{y}$ as candidates for a strict local minimizers of order 1 in the considered optimization problem.

(b) Let $d = 4$.

($b_1$) Suppose that $\bar{y} = (\bar{y}_1, \bar{y}_2)$, where $\bar{y}_1^1 \in ]0, 2[$, $\bar{y}_1^2 = 2 - \bar{y}_1^1$, and $\bar{y}_2 = (1, 1)$. Let $\bar{z} = \bar{y}_1 + \bar{y}_2$. Then

$$\bar{z}^1 + \bar{z}^2 = \bar{y}_1^1 + \bar{y}_1^2 + \bar{y}_2^1 + \bar{y}_2^2 = 4.$$

Hence, it follows from (91) that $P(\bar{z}) = \emptyset$, and the assumption $\operatorname{int}P(\bar{z}) \neq \emptyset$ of Theorem 6 is not satisfied.

($b_2$) – The same as ($a_2$) before.

(c) Let $d = 3$. If $\bar{y}_i \in \operatorname{int}S_i$ for $i = 1$ or $i = 2$, then the respective contingent cone is not pointed and we cannot apply Theorem 6. Suppose now that $\bar{y}_i \in \operatorname{bd}S_i$ for $i = 1, 2$, and take, for example, the point $\bar{y}_1$ such that $\bar{y}_1^1 + \bar{y}_1^2 = 2$, and $\bar{y}_2 = (1, 0)$. Then it is easy to see that $P(\bar{z}) = \emptyset$, and the theorem also cannot be used. On the other hand, if $\bar{y}_1 = (0, 1)$ and $\bar{y}_2 = (1, 0)$, then $K(S_1, \bar{y}_1)+ K(S_2, \bar{y}_2) = \mathbb{R}^2$, and the theorem gives negative answer as in case ($a_2$).

**Discussion** Observe that in case (a) there are no restrictions on the number of cars sold: both factories can sell everything they are able to produce. The results given by Theorem 6 in this case agree with the intuition. In fact, in sub-case ($a_1$), where the maximum production capacity of both factories (i.e., 4 units) is sold, the necessary conditions of Theorem 6 are fulfilled, and so, the corresponding production-consumption plan is a candidate for optimal solution. This plan can be further optimized with respect to the profit of the first factory (while the plan for the second factory remails fixed) but this would require an extended mathematical model taking into account, for example, the prices of cars of class A and B. On the other hand, in sub-case ($a_2$), the production capacity of the second factory is not fully used, and the plan can obviously be improved by increasing $\bar{y}_2^2$ to become 1 (which in fact reduces this case to ($a_1$)). Theorem 6 excludes such plans and this is correct as they are evidently not optimal. Let us now consider case (b). In sub-case ($b_1$) we have the same plans as in ($a_1$) but now the theorem gives no answer because the preference sets are empty. Obviously, each plan with an empty preference set is optimal, and we don't need to apply any optimality conditions here. In case ($b_2$) the situation is identical as in ($a_2$): the theorem excludes such nonoptimal plans. Finally, case (c) is most difficult to handle because Theorem 6 gives no answer if the production plan for some factory belongs to the interior of its production set. But in case of a serious restriction on the number of distributed cars ($d = 3$) it is natural to seek an optimal production plan somewhere between the boundaries of production sets. This shows that better optimality conditions should be developed to include such situations.

## 7 Conclusions

In Sections 2–4, we have presented necessary optimality conditions for strict local minima of arbitrary order for set-valued optimization problems in which minimization is performed with respect to arbitrary preference mappings, not necessarily cone-valued ones. Then, we have applied these results for the special case of order one to welfare economics problems in Section 5 and we have given two concrete examples of such problems in Section 6. However, there are still several points that need attention:

1. Theorem 6 is formulated as a necessary condition for strict local minimizers of order one. It is a rather strong assumption, and one should examine whether or not the same necessary conditions hold for all local minimizers.
2. The examples presented is Section 6 show that, although Theorem 6 can be applied to them, it is not possible at all points. Therefore, developing still better necessary optimality conditions and also sufficient optimality conditions for welfare economics problems with general preference mappings will be important.

**Acknowledgments** The authors would like to thank two anonymous referees for their valuable comments which have greatly helped to improve the previous version of this paper.

**Open Access** This article is licensed under a Creative Commons Attribution 4.0 International License, which permits use, sharing, adaptation, distribution and reproduction in any medium or format, as long as you give appropriate credit to the original author(s) and the source, provide a link to the Creative Commons licence, and indicate if changes were made. The images or other third party material in this article are included in the article's Creative Commons licence, unless indicated otherwise in a credit line to the material. If material is not included in the article's Creative Commons licence and your intended use is not permitted by statutory regulation or exceeds the permitted use, you will need to obtain permission directly from the copyright holder. To view a copy of this licence, visit http://creativecommons.org/licenses/by/4.0/.

## References

[1] Allouch N, Le Van C, Page FH (2006). Arbitrage and equilibrium in unbounded exchange economies with satiation. J. Math. Econ..

[2] Allouch N, Le Van C (2009). Erratum to “Walras and dividends equilibrium with possibly satiated consumers”. J. Math. Econ..

[3] Aubin J-P (1998). Optima and Equilibria: an Introduction to Nonlinear Analysis.

[4] Bao TQ, Mordukhovich BS (2010). Set-valued optimization in welfare economics. Adv. Math. Econ..

[5] Bao TQ, Mordukhovich BS (2014). Necessary nondomination conditions in set and vector optimization with variable ordering structures. J. Optim. Theory. Appl..

[6] Durea M, Strugariu R, Tammer C (2015). On set-valued optimization problems with variable ordering structure. J. Glob. Optim..

[7] Flores-Bazán F, Jiménez B (2009). Strict efficiency in set-valued optimization. SIAM J. Control Optim..

[8] Gale, D., Mas-Colell, A.: On the role of complete, transitive preferences in equilibrium theory. In: Schwödiauer, G. (ed.) Equilibrium and Disequilibrium in Economic Theory, pp. 7–14. D. Reidel Publishing Company, Dordrecht (1977)

[9] Isac G, Khan AA (2009). Second-order optimality conditions in set-valued optimization by a new tangential derivative. Acta Mathematica Vietnamica.

[10] Jahn J (2004). Vector Optimization.

[11] Jahn J, Khan AA, Zeilinger P (2005). Second order optimality conditions in set-valued optimization. J. Optim. Theory Appl..

[12] Khan AA, Soleimani B, Tammer C (2017). Second-order optimality conditions in set-valued optimization with variable ordering structure. Pure and Applied Functional Analysis.

[13] Khan AA, Tammer C (2012). Generalized Dubovitskii-Milyutin approach in set-valued optimization. Vietnam J. Math..

[14] Khan AA, Tammer C (2013). Second-order optimality conditions in set valued optimization via asymptotic derivatives. Optimization.

[15] Khan AA, Tammer C, Zălinescu C (2015). Set-Valued Optimization. An Introduction with Applications.

[16] Li SJ, Sun XK, Zhu SK (2012). Higher-order optimality conditions for strict minimality in set-valued optimization. Journal of Nonlinear and Convex Analysis.

[17] Mas-Collel, A.: Equilibrium theory with possibly satiated preferences. In: Majumdar, M. (ed.) Equilibrium and Dynamics: Proceedings of the Essays in Honour of David Gale, pp. 201–213, MacMillan, London (1992)

[18] Sato N (2000). Satiation and existence of competitive equilibrium. J. Math. Econ..

[19] Studniarski M (2017). Optimization with respect to general preference mappings. Folia Math..

[20] Werner J (1987). Arbitrage and the existence of competitive equilibrium. Econometrica.

[21] Won DC, Yannelis NC (2011). Equilibrium theory with satiable and non-ordered preferences. J. Math. Econ..

